# Developing Psychological Resilience to the Impact of Drought

**DOI:** 10.3390/ijerph20043465

**Published:** 2023-02-16

**Authors:** Matthew Abunyewah, Mitchell K. Byrne, Carol A. Keane, Daniel Bressington

**Affiliations:** Faculty of Health, Charles Darwin University, Darwin, NT 0909, Australia

**Keywords:** drought, resilience, social network analysis, sense of control, social coherence, social connectedness

## Abstract

Background: Drought is a slow-onset natural hazard with significant socioeconomic, environmental and psychological impacts. The extant literature has predominately focused on the physical and economic dimensions of resilience, which mainly address the socioeconomic and environmental consequences of drought. However, the mental health effects of chronic environmental adversity, such as prolonged drought, remain an under-researched area, and frameworks that build and strengthen the psychological aspect of the social resilience of communities are lacking. Methods: This feasibility study will employ a mixed-method design sub-divided into three phases. Phase 1 will utilise social network analysis (SNA) to identify leadership patterns and their intersections across communities. While phase 2 will use semi-structured interviews to ascertain the perceived roles of identified leaders in preparing for and recovering from drought impacts, the third phase will adopt the Delphi method to unpack existing perceptions of control, coherence and connectedness.

## 1. Introduction

### 1.1. Drought and Its Impacts

Drought is a prevalent climate hazard that significantly impacts human lives, livelihoods and property. Evidence from the International Emergency Events Database (EM-DAT) for 2012–2022 shows droughts affected approximately 68.8 million people and inflicted total economic damages in excess of USD 1.1 billion globally [1]). Furthermore, the report demonstrates that drought accounted for over 51% of people affected by disasters and 5% of economic damages from disasters.

While drought is a slow-onset natural hazard, it also produces a complex web of socioeconomic, environmental and psychological impacts that can be either direct or indirect and may extend outside the affected region through to the global level. The extant literature shows that drought directly reduces agricultural production [2,3,4], increases the risk of fire outbreak and severity [5,6,7], aggravates water supply challenges [8,9], causes desertification [10,11,12]) and damages wildlife [13]. The direct consequences via numerous pathways have indirect implications on many facets of human lives, including health [14]. For instance, Kim, Kabir and Ara Jahan [15] found that a lack of freshwater due to drought increases the risk of diseases related to poor health. Elsewhere, food insecurity through drought has been found to indirectly increase psychological distress, anxiety and psychiatric disorders [16,17]. Drought has also been found to directly affect the mental health of farmers [18,19], with studies highlighting an increased risk of suicide as a consequence [20]. Drought stress and concomitant mental health risks extend to the broader affected communities and are not restricted to farmers [19]; however, the mental health effects of chronic environmental adversity, such as prolonged drought, remain an under-researched area [21], and strategies to address potential mental health sequelae have focused on enhancing individual mental health literacy and access to professional support [22,23].

### 1.2. Problem Statement and Research Questions

Projections show an exacerbation in the occurrence and severity of drought in the future in the Northern Territory, Australia [24,25]. For instance, Wang et al. [26] found drought severity increased from 44.26 cm/month to 52.19 cm/month in 2006–2009 and 2018–2020, respectively, and predicted an increased severity due to changes in El Niño and the Indian Ocean Dipole. As a result, there is a growing campaign to build and strengthen communities to foster drought resilience in the Northern Territory. This is because, unlike other disaster hazards such as flooding, drought is ongoing. Strengthening community-based resilience will help communities to be self-sufficient until they receive economic assistance. Studies that have heeded calls to increase communities’ resilience to drought have predominately focused on the physical and economic dimensions of resilience [27,28,29,30]. However, little is known about frameworks that build and strengthen the psychological aspect of the social resilience of communities and buffer against the continuous and evolving nature of drought [31,32]. The absence of psychological resilience frameworks that adequately accommodate the social and psychological risks of drought creates human vulnerability to distress and dysfunction, depression, suicidality, substance abuse and domestic violence [19]. This may lead to a breakdown in social cohesion and connectedness, which diminishes social commitment to long-term drought management. To avert this disconnection and diminution, the current research seeks to strengthen local drought response by developing the adaptive capacities of at-risk communities to deal with the psychological impacts and associated issues following exposure to extreme drought. Specifically, this research has the following aims: (i) identify leadership patterns and intersectionality across communities; (ii) identify perceived potential roles in drought preparedness within leadership and communities; and (iii) investigate the existing perceptions of control, coherence and connectedness of identified community leaders and ascertain the self-identified development needs of disparate agents of community leadership.

### 1.3. Theoretical Underpinnings and Contribution

This project is underpinned by the premise that an individual or community adapts and thrives or experiences crisis and incapacitation based on a perceived sense of control (self-efficacy), feelings of coherence in community response and social connectedness and collective efficacy [33,34]. In line with definitions articulated by Glavin and Schieman [35] and Precht et al. [36], this study defines a sense of control as the concerted belief of communities in their ability to adapt to and respond to drought impacts effectively. Social connectedness implies the idea that individuals are not isolated but rather connected to a specific group or persons [37], while coherence suggests a harmonious alignment of drought adaptation efforts and actions by an individual and the overall community [38,39].

This research makes two major contributions. First, the extant literature has focused mostly on general community resilience, with less focused attention on the psychological component [29,40]. The few studies directly addressing the psychological aspects of resilience have mainly focused on disaster and post-traumatic syndrome as public health implications [41,42,43]. This study contributes to one of the least researched aspects of drought resilience by offering a framework that opens a new discussion on the topic at the community level. Second, to the best of our knowledge, this research is the first to develop a psychological resilience framework for drought in the Northern Territory and Australia as a whole. This framework may also serve as a basis for the establishment of community leadership patterns elsewhere and in response to other adverse events.

## 2. Social Networks and Identification Approaches

A social network encompasses a set of actors and relationships between them, describing communication patterns [44]. Within any social context, it is generally the case that some individuals and groups communicate with each other regularly, others on a less frequent basis, and there are others with whom they may never interact. Identifying social networks facilitates the identification of who communicates with whom and the direct and indirect links or ties between people. Communication links and ties are the terms used to describe the basic units of a social network and are strengthened by the frequency of interactions ([45]). According to Lönnqvist and Itkonen [46], a strong tie is more likely to be formed when there is homogeneity between persons in terms of norms, values, beliefs, socioeconomic status and demographic characteristics. Additionally, Gleave et al. [45] attest that spatial distance is a major determinant of the strength of the ties.

Social networks can be difficult to visualise when the population is large. Wesler et al. [47] and Junquero-Trabado and Dominques-Sal and [48] generally suggest two main stages to assist with this visualisation: (1) knowing and understanding the community to identify roles and (2) creating a role with observed characteristics to classify individuals into pre-defined roles. Furthermore, Gleave et al. [45] propose conceptualisation of social roles into different levels of abstraction. Others have also suggested that identifying roles at the level of social action and descending to a lower level of abstraction to find behavioural regularity is a good starting point [44,45,49]. Zygmunt [44]) summarises the approaches to identifying social roles and networks based on: (i) equivalence classes, (ii) the identification of the core or periphery structure, (iii) the analysis of basic SNA measures and (iv) clustering feature vectors.

For this study, we adopted the analytic approach of basic social network analysis (SNA) because it has demonstrated the capacity to identify influential or central groups and persons in the community capable of influencing the decisions of other community members [49,50,51]. In turn, this may help policymakers and governments target the groups and individuals who are best placed to disseminate information on drought, create drought awareness and lead drought resilience projects in Alice Springs.

## 3. Materials and Methods

### 3.1. Study Design

This study will employ a mixed-method approach involving three phases. Phase 1 utilises social network analysis (SNA) as a primary technique to analyse relations in the community and their characteristics. Social network analysis is an analytic technique that utilises quantitative metrics and visual displays to identify, measure and map social relations within the context of the issue in focus. This project will apply an adapted ego-centric approach to social network analysis (SNA) to identify leadership patterns and intersections across communities, which will focus on the respondent (egos) data in preference to respondents’ ties or social connections (alters) [52,53]. This technique uses graphics with individuals and groups labelled as nodes and relations among individuals and groups denoted as ties, showing strengths and directions [54]. The SNA will be adopted for this study due to its inherent strengths of identifying and analysing patterns of leadership in a complex social structure [55]. The second phase will use semi-structured interviews to ascertain the perceived roles of identified leaders in preparing for and recovering from drought impacts.

The third phase will adopt the Delphi method to unpack existing perceptions of control, coherence and connectedness. The Delphi method is a widely used and recognised technique for aiding decision-making. It involves engaging with experts to solicit and obtain the most reliable consensus of opinion through a series of intensive questionnaires [56,57]. The process is repetitive and maintains participants’ anonymity, controlled feedback and group statistical responses. The Delphi has successfully been employed in several drought resilience studies to analyse drought risk management strategies [58] and identify drought risk vulnerability parameters [59,60]. In this study, the Delphi method will be employed because it is useful in situations with no true or known answers to a phenomenon being studied or investigated, such as long-range forecasting [57,61]. In the case of this research, the sense of coherence, connectedness and control in relation to psychological resilience is unknown, justifying the use of the Delphi method.

### 3.2. Study Settings

Alice Springs is located in the middle of Central Australia’s arid zone. The main source of water supply in Alice Springs is the Roe Creek Borefield and Amadeus Basin, where water is drawn from the Mereenie Aquifer System and the Pacoota Sandstone and Shannon and Goyder Formations. It is home to pastoral and horticulture farming.

**Figure 1:**
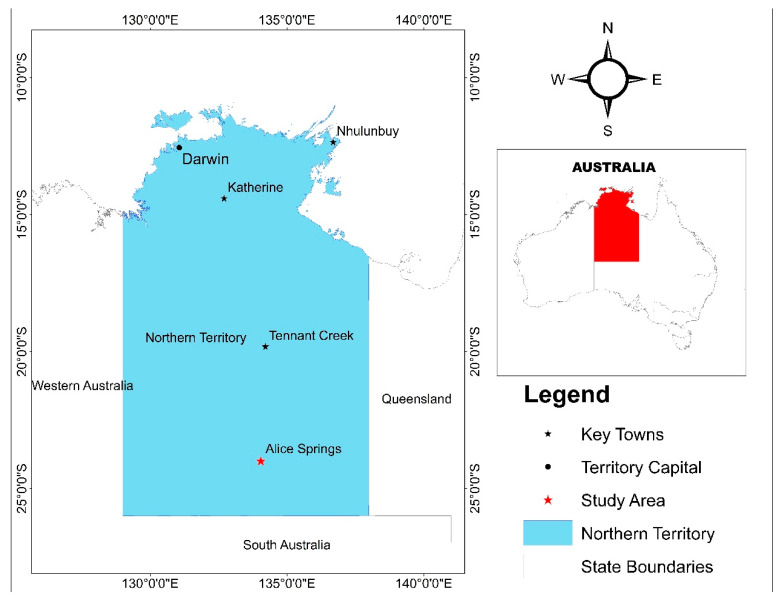
Map showing the study area. Source: Authors’ construct using Google Earth Images, 2023.

Alice Springs is known for its high temperature, low rainfall and drought. According to the Bureau of Meteorology, CSIRO and FarmLink [62], drought is more likely to occur in Alice Springs than in any other place in the NT. The town receives an average of 274 mm of water annually [63]. Over the past 30 years, annual rainfall has increased by 13%; however, available evidence on rainfall reliability between 1989 and 2018 shows that the town has low and unreliable rainfall throughout almost all seasons (Australian Bureau of Meteorology, 2019). In terms of seasonal reliability, summer rainfall has proven to be more reliable than winter. Figure 1 shows the geographical location of the study location.

### 3.3. Participant Inclusion Criteria

Data will be collected from adult residents of Alice Springs who (i) identify as a community member or as being connected to the land and (ii) understand and speak English or, where appropriate, have a trusted relationship with an interpreter and have the capacity to provide informed consent.

### 3.4. Sample Size

The study will be divided into three phases, with each phase having a different sample size. The debate on sample size adequacy for SNA is still in its infancy. As a result, the sample size for phase 1 of this study was determined by taking average sampled SNA studies utilising SNA as an analytical technique. In the first phase of the study, a minimum sample size of 50 will be required [64]. In addition, while the sample size for the second phase will depend on the outcome of phase 1, that of the third phase will also depend on phase 2.

## 4. Data Collection and Analysis

### 4.1. Phase 1

The ego-centric SNA in phase 1 will employ questionnaire surveys and structured interviews to gather information on leadership structures and patterns in Alice Springs. This approach will focus on data collected from respondents (egos) without necessarily engaging respondents’ ties or social connections (alters).

#### 4.1.1. Interview Guide

To answer the first research aim, engagement with community members will focus on leadership identification, selection and patterns in Alice Springs and their interconnectedness. These questions are underpinned by Boone et al.’s [65] leadership approaches: positional, reputational, opinion leadership, decision-making and social reputation. Adopting and modifying questions from Van Den Ban’s [66], participants will be asked:

“*Which people do the community look to for information and advice and why?*”

“*Who does the community look to when an important decision needs to be made and why?*”

“*Who are the most influential people in this community, and why?*”

#### 4.1.2. Network Data Collection

Prior to data collection, the researchers will conduct a reconnaissance survey to become familiar with the topography, boundaries and other physical characteristics of Alice Springs. This will allow the researchers to meet up with the leaders of industry associations, community brokers and traditional leaders who can potentially assist the team in building a good relationship with the study community. Preliminary engagement with community leaders will also help to discuss the research project and bring together a local project reference group. The reference group will inform the co-construction of the project and provide ongoing critique and feedback at every stage of the research.

A modified stratified sampling technique will be adopted to recruit participants for the study. Alice Springs will first be divided into several strata (e.g., industry peak body, education council, sports council, community-based associations, etc.). A meeting will be organised between the research team and the heads of these organisations to introduce the research team, explain the study’s purpose, and assure them that the study’s outcome will be used to support community preparedness for drought and academic purposes. The research team will also provide copies of the participant information sheet and consent form to the heads of the selected institutions. An appointment will be made (face-to-face or online) to meet the full membership of the selected organisation/community and explain to them the purpose of the study and the inclusion and exclusion criteria. Only participants who demonstrate an understanding and provide either verbal or written agreement with the participant information sheet and consent form will be included in the study. Respondents in this study will be selected using simple random and snowball sampling techniques. The snowballing sampling technique will be used as a supplementary method to recruit further respondents [67].

#### 4.1.3. Network Data Analysis

The SNA technique will be employed to answer the first research aim—to identify leadership patterns and intersections across communities. The SNA as a technique is underpinned by the assumption that social life is created primarily by relations and the networks formed through relations [68,69]. It focuses on structural relationships such as kinship, defined role relationships (e.g., friends) and affective ties among individuals and groups in the community [68]. The SNA will be employed to identify leadership patterns and their intersections across communities because the technique has inherent strengths in identifying formal and informal relationships and what facilitates or impedes information flows that bind the ties and networks [70,71,72].

Network data collected from the study will be analysed using Gephi Network Analysis software. The network data will be analysed to check for centrality (degree, betweenness and closeness), structural equivalence and density to answer the first research aim. The degree of centrality will be identified using the number of nominations; thus, the higher the nomination, the higher the in-degree centrality [73]. Furthermore, betweenness centrality, which refers to the influence of nominated leaders over the flow of information among groups, will be computed [74,75]. In other words, betweenness centrality will identify leaders who will serve as a gateway to information flow in the network. Additionally, closeness centrality will be computed to identify nominated leaders who can reach the most people in a given range of steps [49]. Structural equivalence analysis identifies the extent to which nodes have a common linkage [76]. Findings from this study will serve as a basis to investigate the perceived potential roles of the identified influential leaders in drought preparedness (Phase 2).

### 4.2. Phase 2

The SNA result will provide data on the community’s most influential and trusted individuals/leaders relevant to developing adaptive community capacity. Using semi-structured interviews, the leaders identified through the SNA will be interviewed to ascertain their perceived potential roles in preparing for and recovering from drought impacts. This will be completed based on the respondents’ preferred choice of medium of engagement—face-to-face, telephone or online. Interviews will be recorded using a digital audio recorder, or interpreters will be engaged where necessary. Data obtained from the field will be edited, transcribed verbatim and transferred into NVIVO software. Thematic content analysis will be utilised to organise the data and find ideas, patterns and concepts about the perceived potential roles of the identified leaders in drought preparedness. Results from this phase will also support the development of content to investigate the existing leaders’ perceptions of control, coherence and connectedness, including the sources of these beliefs and their applicability to future drought conditions, using the Delphi research method (Phase 3).

### 4.3. Phase 3

A 3–4 round Delphi process will engage identified leaders in each community to unpack existing perceptions of control, coherence and connectedness. Each round of engagement will consist of identified leaders purposively selected. The Delphi approach will help build a consensus on what constitutes a sense of coherence, connectedness and control. Given that drought is a catchment-wide problem that can intersect several communities, this will include data collection within specific communities and between communities and will be based on their in-depth knowledge of drought preparedness and management, including understanding community practices and local governance. Prior to the engagements, the research team will undertake an in-depth literature review to develop a draft Delphi format and statements. The Delphi survey will be conducted to refine the Delphi statements and gain insight into the community’s perceptions of control, coherence and connectedness. Descriptive statistics and thematic content analysis will be used to analyse the data gathered.

### 4.4. Ethical Approval

The Charles Darwin University Human Research Ethics Committee has approved this research (ref no. H22092). As part of the ethics approval, only participants who read/have been read to and indicate a clear understanding of the participant information sheet and consent form and either sign or provide verbal consent will be included in the study. Where the respondent gives verbal consent, the researcher will sign and document the date.

## 5. Discussion

Recently, the urgency and increasing impacts of drought have called for an accelerated, coordinated partnership among all stakeholders. The International Panel on Climate Change [77] suggests that developing locally relevant, culturally appropriate and sustainable solutions for drought requires communities to lead with adequate support from international agencies, national governments, experts and non-governmental organisations. This also requires strengthening and empowering community networks [78,79]. Supporting this point, Favero and Sarriera [80] indicate that community knowledge and skills need to be evaluated and enhanced for local people to take the central stage of developing sustainable drought initiatives.

The way drought risk is understood, and people’s views on how it might develop and change across different communities are essential. Each community/locality requires strategies tailored to its specific current and anticipated population dynamics, circumstances and characteristics. Thus, the current project will focus on identifying communities, their members, and their leadership structures in the Alice Springs area. Findings from this research will provide foundational knowledge about how to develop a sustainable drought resilience program at the community level across the Northern Territory. This will support communities to proactively prepare for and adapt to the continuous, dynamic and evolving psychological and social impacts of drought.

This research contributes to the community resilience literature by identifying leadership patterns and intersections across communities in the Northern Territory. Building the sense of control of residents of Alice Springs is expected to increase people’s confidence in their ability to respond to the evolving nature of drought. This conforms with other studies that have found a positive relationship between self-efficacy and climate adaptation [34,81,82]. In addition, identified networks may be used to increase the connectedness or cohesiveness of the residents of Alice Springs. This may help enhance drought information dissemination, preparedness and response. It is also expected that a highly cohesive Alice Springs may help identify collective drought resilience goals and facilitate their implementation to achieve set targets [83,84,85].

Furthermore, this research will complement and enhance the Federal and Northern Territory Governments’ vision of raising drought awareness and strengthening local community capacities to plan and prepare for drought and better adapt to future drought. The results will be relevant to other jurisdictions’ planning and response to adverse events. At the international level, this research will contribute to the achievement of the United Nations Sustainable Development Goals 6 (Ensure availability and sustainable management of water and sanitation), 11 (Make cities and human settlements inclusive, safe, resilient and sustainable), 13 (Take urgent action to combat climate change) and 17 (Strengthen the means of implementation and revitalise the global partnership for sustainable development) at the community level.

## 6. Conclusions

It is widely recognised that drought has significant economic and social impacts on affected communities; however, less is known about the psychological impacts of drought—including best-practice approaches to build and strengthen the psychological resilience of communities. This research seeks to address this knowledge gap through development of a novel structured, community-empowering framework and process for building psychological resilience of drought-affected communities. Adopting a co-design phase-based methodological approach, leadership patterns and intersections across communities will be identified using social network analysis. The perceived roles of these identified leaders in preparing their communities for drought and drought recovery will then be explored through semi-structured interview. Lastly, the Delphi method will be utilised to unpack existing perceptions of control, coherence and connectedness in relation to psychological resilience within the community in focus. This research will contribute significantly to both theory and practice of drought preparedness and response through development of a co-design informed framework that i) identifies communities’ psychological resilience needs, and ii) brings together communities and their leaders to adapt to develop interventions to recover from the psychological and social impacts of drought. It is anticipated that the framework will have translational reach for drought-affected communities across the nation.

## Data Availability

Not applicable.
